# Influence of Practice Periodization and Sleep Duration on Oxidative Stress in High School Judo Athletes

**DOI:** 10.3390/sports11090163

**Published:** 2023-08-30

**Authors:** Ena Yoshida, Harumi Hayashida

**Affiliations:** Graduate School of Sport Sciences, Toin University of Yokohama, 1614 Kuroganecho, Aoba Ward, Yokohama 225-0025, Japan; grazia.e25@gmail.com

**Keywords:** junior athlete, oxidative stress, sleep, periodization, judo

## Abstract

Numerous research studies have investigated the relationship between exercise, oxidative stress level, and condition in athletes who engage in intense training on a daily basis. However, it is known that oxidative stress is affected by exercise, sleep, and the psychological state, but there are only a few studies that have comprehensively examined oxidative stress based on the actual practice periods and living conditions of athletes. Therefore, our study aimed to explore the influence of three distinct training periods (short training period, intensive training period, and pre-competition periods) as well as life situations (sleep and number of steps) on oxidative stress levels (diacron reactive oxygen metabolites: d-ROMs) in high school judo athletes. The results showed that, among the three periods, the level of oxidative stress increased the most during the pre-competition period, and the value was higher than during the training period, when the intensity of training was highest. The levels of the d-ROMs values during the pre-competition period were negatively correlated with the amount of sleep on the previous day. The findings suggest that, besides the exercise intensity, factors such as sleep duration and other life situations should be regarded as critical considerations for high school judo athletes.

## 1. Introduction

When oxygen is utilized for energy metabolism in the human body, a portion of it undergoes a transformation into reactive oxygen species (ROS), such as superoxide, hydrogen peroxide, and hydroxyl radicals. ROS are inherent byproducts of cellular metabolism and serve vital roles in diverse physiological processes, encompassing cell signaling [1] and immune responses [2]. However, an excessive of ROS can induce cellular damage and contribute to a spectrum of health issues, including chronic diseases and the aging process [3]. The body has the antioxidant capacity to protect cells, DNA, and other tissues from ROS; when the production of ROS surpasses the body’s antioxidant capacity, it leads to a state known as oxidative stress. Generally, exercise, accompanied by increased oxygen intake, is known to increase oxidative stress. Repeated exposure to exercise-induced increased oxidative stress causes the organism to develop an adaptive response and exert an adaptive antioxidant capacity in exercise and in oxidative environments unrelated to exercise [4]. Certain ROS are also important for skeletal muscle augmentation [5,6], and such hormesis effects are important for athletes. Conversely, reactive oxygen species act as mediators in inflammatory responses [7], and excessive conditions can lead to muscle contraction dysfunction, weakness, and fatigue [8]. Recent evidence suggests that this is associated with overtraining syndrome [9]. Therefore, for athletes, maintaining an optimal oxidative stress state is considered important for performance.

Numerous studies have confirmed that the factors that cause oxidative stress are not limited to exercise, but involve a wide range of factors, including sleep, diet, age, disease status, and psychological state. The same is true for antioxidant capacity. Therefore, in order to closely examine oxidative stress in actual athletes, and use it to improve performance, it is necessary not only to focus on the adjustment of exercise intensity, but also to investigate their living conditions as well. Among other things, it has been reported that insufficient sleep is a risk factor for poor performance [10] and injury [11]. Sleep is considered important for athletes to recover from fatigue and maintain physical and mental condition, but no studies have investigated the relationship between oxidative stress and sleep in athletes. In addition, this is especially important in high school athletes, the age at which the adjustment of intensity, duration, and frequency of practice becomes important in order to improve competition-specific strength, power, and technique [12,13]. Therefore, a detailed investigation of how practice phasing, practice intensity, and living conditions affect the oxidative stress status would contribute to the development of more effective conditioning methods. Additionally, competitions involving weight control have also raised concerns that weight loss in preparation for competitions can exacerbate oxidative stress [14].

Against this background, the study focused on judo, a sport that has been noted for its high frequency of injuries [15,16] and which is popular worldwide [17]. The subjects selected were high school athletes, and the objective was to investigate the impact of three practice periods, daily life activities, and sleep on oxidative stress.

## 2. Materials and Methods

### 2.1. Subjects

Subjects were nine high school female judo athletes belonging to the same club. They had 8–13 years of judo experience and consistently competed in national judo tournaments for high school students. The age of the subjects was 16.3 ± 0.5 years. The subjects were free of infection before and after the measurement period, participated in all practices, and were scheduled to compete the day after the pre-competition practice period.

### 2.2. Measurement Period and Measurement Items

**Figure 1:**
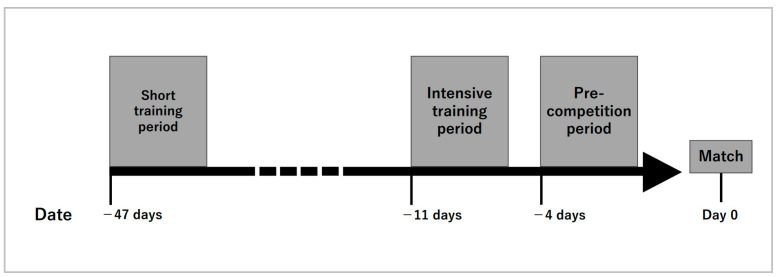
Comprehensive overview of the measurement period.

The measurement period spanned from November 2022 to January 2023 (Figure 1). The survey was conducted on four consecutive days during each of the following periods: (1) Short training period: a period when students had a normal commute to school and practiced freely with shortened hours; (2) Intensive training period: a reinforced practice period during the school winter break; (3) Pre-competition period: a tapering period before the day before a match.

Body weight and body fat percentage were measured on the first day of each period. Measurements were taken in the early morning before exercise on an empty stomach using the Omron Body Weight Composition Analyzer KaradaScan HBF-371 (Omron Healthcare Co., Ltd., Kyoto, Japan).

The activity survey was conducted using both step counts and a survey form. The number of steps was recorded from waking on the first day of each study period until bedtime on the third day. Additionally, the survey form was completed up to waking on the fourth day. The number of steps (the number of vibrations captured by the sensor) was recorded using Lifecorder EX (Suzuken Co., Ltd., Nagoya Aichi, Japan). Lifecorder is a small (72.5 × 41.5 × 27.5 mm) and lightweight (60 g) activity meter. It can store the number of daily steps for several days. Note that the Lifecorder EX was removed during practice time, bathing time, and sleeping time. The survey form requested participants to chronicle all activities, including sleeping hours, specifying time and details.

Blood samples were collected every morning (before breakfast from 6 to 9 a.m. and before exercise) on days 2–4 of the study. The blood collection was conducted by the subjects themselves. Using a blood-collection puncture device (Wismer, Inc., Waterloo, ON, Canada), a small amount of blood was collected into a heparin-coated blood-collection tube (Wismer, Inc.) by puncturing the fingertip. The amassed blood underwent centrifugation and subsequent measurement to assess oxidative stress (diacron reactive oxygen metabolites: d-ROMs) and antioxidant capacity (biological antioxidant potential: BAP) employing the REDOXLIBRA (Wismer, Inc.).

One of the markers of oxidative stress level is d-ROMs. It is a comprehensive indicator of metabolites produced when reactive oxygen species and free radicals oxidize lipids, proteins, amino acids, and nucleic acids in the body. It measures mainly hydroperoxide (ROOH) in blood using a color reaction [18]. The BAP test is an indicator of the ability to reduce trivalent iron ions in a sample to divalent iron ions [18]. Due to their susceptibility to diet and exercise, blood samples were collected in this study on an empty stomach before exercise. Additionally, the d-ROMs test results are increased by events that induce oxidative stress [19]. To minimize the impact of daily variation in individuals, the average value was calculated from measurements taken over three days in each period and used as the representative value for each individual in that period.

### 2.3. Statistical Processing

All measurements are presented as means ± standard deviation. The Kolmogorov–Smirnov test was performed to evaluate the normality of the data. Since the test results showed no normality in any of data, the Friedman test was used to examine the differences among the three variables (short training period, intensive training period, pre-competition period). The items for which significant differences were found were examined using Wilcoxon’s multiple comparisons. Pearson’s correlation analysis was conducted to explore the relationships between variables. The significance level was set at *p* < 0.05 for all analyses. Statistical processing software (IBM SPSS Statistics Ver. 28.0.1.0) was used for statistical processing.

## 3. Results

### 3.1. Body Composition of Subjects during Each Petiod

Subjects’ height was 160.6 ± 4.8 cm. There were no significant differences in body weight and body fat among any of the time periods (Table 1).

### 3.2. Physical Activity Record

#### 3.2.1. Practice Content and Intensity

Based on practice records and team practice menus, the practice content and intensity during the study period (days 1–3) are as outlined below.

The average practice time per day during the “short training period” was two hours. The practice consisted of about 30 min of morning practice and 1.5 h of afternoon practice. Practice time was the shortest among the three periods, and was of low intensity. Practice consisted mainly of short physical training and confirmation of judo techniques.

The average practice time per day during the “intensive training period” was 5.5 h. On the day before blood collection, the first and second days consisted of two sessions of 3 h in the morning and 3 h in the afternoon, and the third day was a single session of 4 h in the morning. The main contents of practice were “uchikomi”, which is the practice of repeating the process of throwing an opponent on the spot or while moving, and “randori”, which is free practice in which both parties apply techniques to each other, and is the most practical practice method that reflects the competitive state of the match in practice.

The average practice time per day during the “pre-competition period” was 2.6 h. There were no morning practices. Afternoon practice consisted mainly of judo “uchikomi” and “randori”. The intensity was reduced from normal practice.

#### 3.2.2. Sleep Times

**Figure 2:**
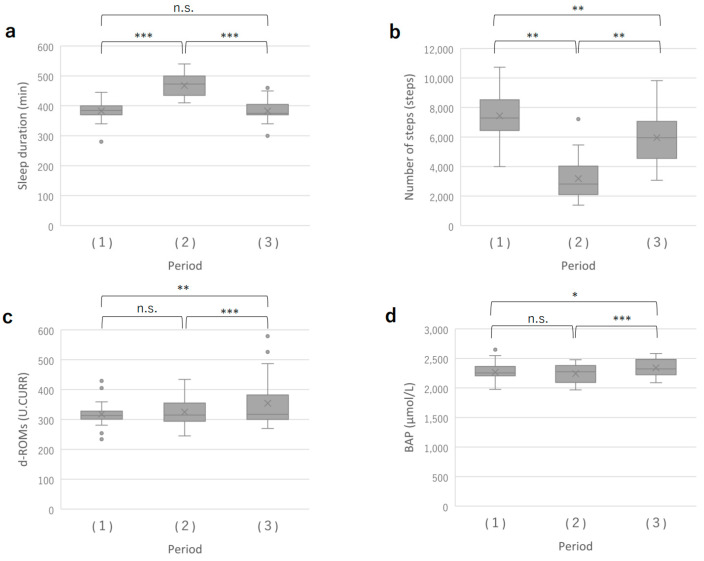
Comparison of sleep duration, steps, d-ROMs, and BAP over 3 time periods. (**a**) Sleep duration; (**b**) Steps; (**c**) d-ROMs value; (**d**) BAP for 3 different practice periods. (**1**) Short training period; a period when students had regular commuting to school and practiced freely with shortened hours. (**2**) Intensive training period; a period of reinforced practice during the school winter break. (**3**) Pre-competition period; a tapering period before the day before a match. *: *p* < 0.05 **: *p* < 0.01 ***: *p* < 0.001. n.s.: Not Significant.

The average sleep duration over the three days was 383 ± 30 min during the short training period, 467 ± 38 min during the intensive training period, and 382 ± 36 min during the pre-competition period. The sleep duration was significantly longer during the intensive training period (Figure 2a).

#### 3.2.3. Physical Activity

The average number of steps over the three days was 7440 ± 1820 during the short training period, 3188 ± 1436 during the intensive training period, and 5951 ± 1768 during the pre-competition period. The number of steps significantly decreased in the order of short training period, pre-competition period, and intensive training period (Figure 2b).

### 3.3. Oxidative Stress and Antioxidant Capacity in Blood

The levels of oxidative stress (d-ROMs) were found to be significantly higher during the pre-competition period compared to both the short training period and the intensive training period. There was no significant difference was observed between the short training period and the intensive training period (Figure 2c).

Similarly, the antioxidant capacity (BAP) was also significantly higher during the pre-competition period compared to both the short training period and the intensive training period. There was no significant difference between the short training period and the intensive training period in terms of antioxidant capacity (Figure 2d).

### 3.4. Relationship between Sleep Duration and Oxidative Stress

**Figure 3:**
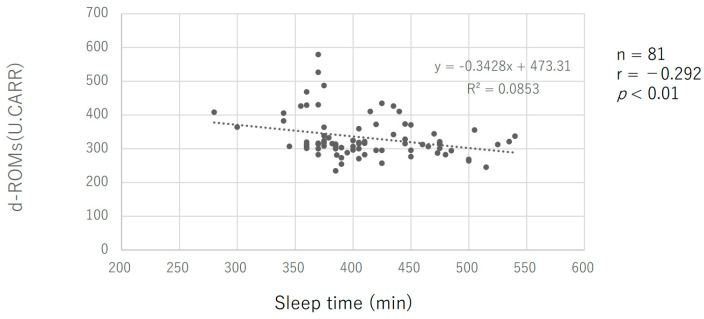
Relationship between sleep duration and oxidative stress.

There was a negative correlation between sleep duration (min) and the oxidative stress level (d-ROMs), with a correlation coefficient of −0.292 (*p* = 0.008) (Figure 3). There was a negative correlation between the antioxidant capacity (BAP) and sleep duration (min), with a correlation coefficient of −0.298 (*p* = 0.009).

### 3.5. Relationship between d-ROMs and BAP

**Figure 4:**
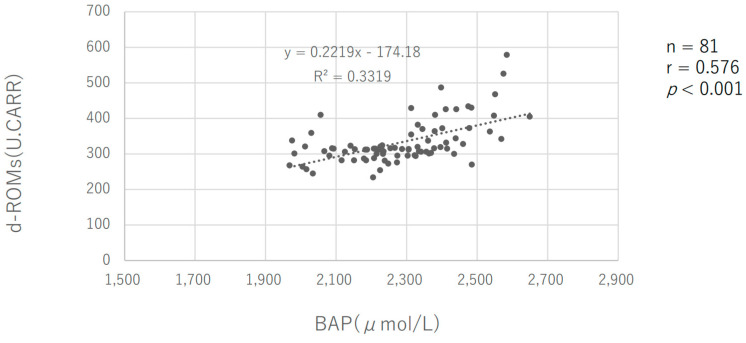
Relationship between d-ROMs and BAP.

There was a positive correlation between the d-ROMs and BAP, with a correlation coefficient of 0.576 (*p* < 0.001) (Figure 4).

## 4. Discussion

In this study, we examined the impact of three practice periods, daily life activities, and sleep on oxidative stress in high school female judo athletes. The primary finding of our investigation revealed that the oxidative stress level of these athletes was significantly higher during the pre-competition period compared to the intensive training period, which involved the highest exercise intensity among the three periods (short practice, strengthening practice, and pre-competition practice). Interestingly, our results suggest that the oxidative stress level in high school female judo athletes might be more influenced by their sleep duration than the intensity of exercise on the previous day.

The validity of the practice intensity and living conditions during each period can be inferred from the records of practice time and content, as well as the number of steps taken. In a prior study [20], it was reported that skilled female judo practitioners exhibited heart rates ranging from 174 ± 1.4 to 182 ± 3.3 bpm during “randori”, and from 163 ± 9.2 to 176 ± 4.3 bpm during “uchikomi”. In addition, it is also reported that the most intense part of judo practice is “randori”, followed by “uchikomi” [21]. Therefore, the intensive practice period in which these two practice menus, which are considered high intensity, were performed for a long time is the most intense of the three practice periods. And during the study period, participants wore the Lifecorder, except during practice time, bathing time, and sleeping time. The fact that the number of steps decreased in the order of short training period, pre-competition period, and intensive training period may reflect the fact that the intensive training period included longer practice time, more sleep time, and no school events. Furthermore, the number of steps taken during the pre-competition period was lower than during the short training period, despite the fact that the pre-competition period and the short training period had the same sleep duration and the same commute to school. This can be inferred to indicate a difference in training time and intentional resting (tapering) before the match.

In this study, oxidative stress levels were found to be most elevated during the period of reduced practice intensity and tapering, which is typically undertaken in preparation for a match. This suggests that the pre-competition conditions may have contributed to increased oxidative stress. We believe that the causes reflect weight loss and psychological factors. First, judo is a sport that requires weight control, and rapid weight loss is often implemented just before a match [22]. In a study of 20 male wrestlers, Yanagawa et al. (2010) showed that rapid weight loss significantly increased urinary 8-OHdG levels, an oxidative stress marker, in the subjects [23]. Moreover, Nishimaki et al. (2017) reported that rapid weight loss significantly increased blood d-ROMs levels in nine male wrestlers [14]. Rapid weight loss typically includes methods such as sauna-suit usage and restriction of water intake several days before a match, leading to temporary weight loss. This practice has been suggested to induce increased oxidative stress due to thermal and osmotic loading [24]. In this study, blood samples were taken under conditions immediately prior to the match, and in three of the nine subjects, weight adjustment or rapid weight loss for the match actually had been performed. Hence, it is highly likely that weight loss had an impact on the oxidative-stress-level data. Secondly, the pre-match psychological state may have also affected the data. Hayashi (2015) and Daniela (2022) demonstrated that psychological stress may increase oxidative stress through hypothalamus–pituitary–adrenal activation and stress endocrine responses, such as sympathetic excitation and parasympathetic inhibition, causing autonomic imbalance [25,26]. Moreover, Hisamitsu et al. (2017) reported in a study on rats aimed at developing an experimental apparatus for psychological stress that mental stress and associated autonomic nervous system disturbances increase blood d-ROMs in rats [27]. In a human study, Sugiura et al. (2022) reported that, in basketball players preparing for a competition, d-ROMs values were higher in regular players immediately before the competition, and a significant increase in d-ROMs values was observed, especially in players in positions that are considered to be physically and mentally demanding [28]. Based on these previous studies, it is possible that the particular mental situation prior to the competition may have affected the level of oxidative stress in the subjects in this study.

Many studies on the relationship between exercise and oxidative stress have reported that both aerobic and anaerobic exercise increase oxidative stress biomarkers after exercise [29,30,31,32], and it is clear that acute exercise causes oxidative stress to the body [33]. Furthermore, oxidative damage has been shown to escalate with exercise intensity [34] and duration [35]. For instance, intense exercise, such as a full marathon, leads to a significant production of reactive oxygen species and elevated oxidative stress levels for several days after the race [36]. Additionally, oxidative stress is increased during training camps, particularly during consecutive days of intense exercise, in comparison to normal training [37]. Given the existing literature, our initial assumption was that, among the three training periods, the oxidative stress level would be higher during the intensive training period, where the exercise duration is longer, and the intensity is higher. Surprisingly, however, the oxidative stress level was found to be the lowest during the intensive training period. The lower oxidative stress during this period may be attributed to the following two factors.

The first possibility is that the stimulation by reactive oxygen species generated by intense exercise may have resulted in greater activation and continued enhancement of antioxidant capacity. In a study conducted by Nagashima (2011), during exercise performed by well-trained cyclists on a bicycle ergometer (60% VO_2_max), no significant difference in d-ROMs were found at five time points: before exercise, 1 h after the start of exercise, 2 h after the start of exercise, 3 h after the start of exercise, and 1 h after the end of exercise. However, the BAP continued to increase with the duration of exercise, and remained significantly increased until 1 h after the end of exercise [38]. Moreover, in studies of antioxidant enzyme activity in rat muscle tissue [39,40], it has been reported that superoxide dismutase (SOD) activation induced by exercise is conditional on longer exercise duration and higher exercise intensity. This indicates that certain antioxidant enzymes are more active when exercise duration and intensity exceed certain thresholds. Based on these findings, it is possible that during the intensive training period, when the exercise intensity was the highest and the duration of exercise was the longest, the antioxidant system of the body, including antioxidant enzymes, was more strongly activated than during the other two periods, resulting in a reduction of oxidative stress the morning after exercise.

Secondly, the sleep duration of the subjects in this study showed that they slept the longest during the intensive training period (Figure 2a). On all measurement days, there was a negative correlation between d-ROMs values and the previous day’s sleep time (Figure 3). The longer the sleep time, the lower the oxidative stress level, suggesting that the extended sleep duration may have contributed to the reduced oxidative stress level. The longer sleep duration of the subjects during the intensive training period could be attributed to having more time in their daily schedule compared to when they had to commute to school due to long school vacation periods. Additionally, it is known that d-ROMs values correlate with BAP values in previous studies. This was also the case in the present study (Figure 4). Thus, BAP, which indicates antioxidant capacity, was negatively correlated with sleep duration, and BAP decreased with longer sleep duration, which may be largely due to the marker’s characteristic that d-ROMs shows a BAP correlation. We hypothesize that this sleep duration played a major role in ROS scavenging. Although the mechanism of sleep’s effect on oxidative stress is not yet fully understood [41], there is a close relationship between adequate nighttime sleep and melatonin secretion, a hormone with powerful antioxidant properties [42,43]. One previous study showed that, in healthy adult men, a night of sleep deprivation increased DNA methylation due to oxidative stress caused by decreased glutathione, a major intracellular antioxidant [44]. In addition, Gulec et al. (2012) compared the blood of insomnia sufferers and controls and reported significantly higher levels of oxidative stress as measured by malondialdehyde (MDA), a marker of lipid peroxidation in the blood of insomnia patients [45]. Furthermore, in a study by Chen et al. (2022), who observed the blood of healthy male physicians after one night of night-shift work, they observed that malondialdehyde (MDA) was significantly elevated after night-shift work compared to baseline [46]. Based on these previous studies and conditions, it is likely that adequate sleep helped to reduce oxidative stress generated during the day in the present subjects.

ROS produced by exercise cause favorable adaptations, such as increased antioxidant capacity and muscle hypertrophy, due to the hormesis effect. However, excessive ROS promote inflammation and cause fatigue and other detrimental phenomena. Although it is considered important to control the balance between these things as they relate to the physiological performance of athletes, it remains controversial whether exercise-induced increases in oxidative stress have detrimental effects on long-term physiological function [33]. The present study suggests that maintaining approximately 8 h of sleep can help balance oxidative stress at the field level in high school athletes. This duration of sleep may prevent the continuation of damage caused by ROS into the next day, while still allowing for the training effects of ROS produced by intense exercise. The results also highlighted the importance of considering sleep duration over exercise intensity in avoiding the risk of excessive oxidative stress in athletes.

Athletes are often under strain, implementing weight loss along with the intensity of daily practice. Additionally, high school athletes are expected to have a demanding schedule due to classes and studies, and when commuting to and from school, they are likely to have difficulty obtaining adequate sleep. These factors should be fully recognized by instructors. In the case of high school athletes, it is crucial for adults to provide appropriate conditioning guidance and adjust the quantity and quality of practice based on the situation. Moreover, proper weight control should be based on expert opinion.

To date, no studies have focused on the effects of practice periodization on oxidative stress in high school judo athletes. Moreover, no studies that have simultaneously examined the dynamics of oxidative stress in athletes under exercise conditions and sleep. Therefore, the present study will have implications not only for high school judo athletes but also for high school athletes, where the separation of training periods is gaining importance, as well as for weight-control competitions and female athletes.

This study has several significant limitations. The sample size of nine subjects is relatively small, which may limit the generalizability of the findings to all high school judo players. Despite this limitation, the insights gained from this study can serve as a valuable foundation for designing future large-scale studies with a larger and more diverse sample. Dietary content during the measurement period was ascertained by photography, and within subjects, there were no significant changes in the dietary environment, except for the pre-competition period of those who lost weight, but detailed dietary nutrients were not calculated. Additionally, this study focused on female athletes who were high school students, and detailed analysis of menstrual cycles was not included due to the nature of the study being conducted on high school students. Considering the suggestion that the female menstrual cycle may influence the redox response [47], future studies should carefully consider the effects of the menstrual cycle when collecting data from female athletes.

## 5. Conclusions

We investigated the effects of three practice phases (short training period, intensive training period, and pre-competition period) on oxidative stress in high school female judo athletes. The results demonstrated that oxidative stress was highest during the pre-competition period, indicating a potential relationship with sleep duration. Besides considering exercise intensity, it is crucial to give special attention to sleep duration in conditioning female high school judo athletes.

## Figures and Tables

**Table 1:** Subjects’ body weight and body fat percentage for each measurement period.

Measurement Period	Body Weight (kg)	Body Fat Percentage (%)
short training period	66.9 ± 13.6	26.2 ± 5.4
intensive training period	67.0 ± 14.7	26.6 ± 5.1
pre-competition period	66.0 ± 14.7	26.2 ± 5.6

## Data Availability

The data associated with the article are not publicly available because they contain information that could compromise the privacy/consent of the research participants, but can be obtained from the corresponding author upon reasonable request.
